# Lagrangian Split-Step Method for Viscoelastic Flows

**DOI:** 10.3390/polym16142068

**Published:** 2024-07-19

**Authors:** Martina Bašić, Branko Blagojević, Branko Klarin, Chong Peng, Josip Bašić

**Affiliations:** 1 Faculty of Electrical Engineering, Mechanical Engineering and Naval Architecture, University of Split, R. Boškovića 32, 21000 Split, Croatia; bblag@fesb.hr (B.B.); bklarin@fesb.hr (B.K.); jobasic@fesb.hr (J.B.); 2 Engineering Software Steyr, Berggasse 35, 4400 Steyr, Austria; chong.peng@essteyr.com

**Keywords:** viscoelasticity, polymers, Oldroyd-B, meshless, LDD, sudden contraction, die swell

## Abstract

This research addresses and resolves current challenges in meshless Lagrangian methods for simulating viscoelastic materials. A split-step scheme, or pressure Poisson reformulation of the Navier–Stokes equations, is introduced for incompressible viscoelastic flows in a Lagrangian context. The Lagrangian differencing dynamics (LDD) method, which is a thoroughly validated Lagrangian method for Newtonian and non-Newtonian incompressible flows, is extended to solve the introduced split-step scheme to simulate viscoelastic flows based on the Oldroyd-B constitutive model. To validate and evaluate the new method’s capabilities, the following benchmarks were used: lid-driven cavity flow, droplet impact response, 4:1 planar sudden contraction, and die swelling. These findings highlight the LDD method’s effectiveness in accurately simulating viscoelastic flows and capturing large deformations and memory effects. Even though the extra stress was directly modeled without any regularization approach, the method produced stable simulations for high Weissenberg numbers. The stability and performance of the the Lagrangian numerics for complex temporal evolution of material properties and stress responses encourage its use for industrial problems dealing with polymers.

## 1. Introduction

Many synthetic and natural fluids exhibit complex rheological behavior, with viscoelasticity being an important fluid property. The accurate prediction of viscoelastic flows is critical for a broad range of industrial and biological applications, such as fluid dynamics and materials science. Complex behaviors of fluid with elastic properties are encountered in processes from polymer manufacturing to physiological systems. This research will use a meshless and Lagrangian method to simulate viscoelastic flows, incorporating the Oldroyd-B constitutive model that is well-known for its ability to capture complex viscoelastic behavior. As industries increasingly rely on numerical simulations to optimize processes and predict material behavior, the development of robust computational tools becomes critical. The Lagrangian meshless methods offer a promising approach for accurately and efficiently capturing the dynamics of viscoelastic flows. This study aims to address the current knowledge gaps in numerics of Lagrangian meshless methods, with the goal of providing insights that contribute not only to the fundamental understanding of viscoelastic phenomena but also to the optimization and innovation of processes in fields as diverse as materials engineering and biomedical sciences.

Continuum mechanics is the most frequently employed approach for modeling macroscopic flows. In order to obtain continuum constitutive equations from kinetics theories, closure approximations are necessary. Still, in practical numerical simulations, the constitutive model and material characteristics are typically derived from rheological measurements. Nevertheless, it is important to approach the utilization of constitutive models derived from curve-fitting of rheometric flows (such as simple shear or uniaxial extensional) to more complex flows with careful consideration [1]. Because of their complex behavior, viscoelastic flows, which are characterized by the interaction of viscous and elastic effects [2], pose challenges in numerical modeling. Viscoelastic flows, which involve the interaction of viscous and elastic effects, present difficulties in numerical modeling due to their intricate behavior. Because of the stiff hyperbolic form of the differential constitutive equations, computational modeling of viscoelastic flows is susceptible to both numerical instabilities and physical flow fluctuations, as well as nonphysical constitutive instabilities [1,3]. The numerical challenge of solving these constitutive equations, referred to as the high Weissenberg number problem (HWNP), has been a significant barrier in computational rheology for over twenty years.

**Figure 1:**
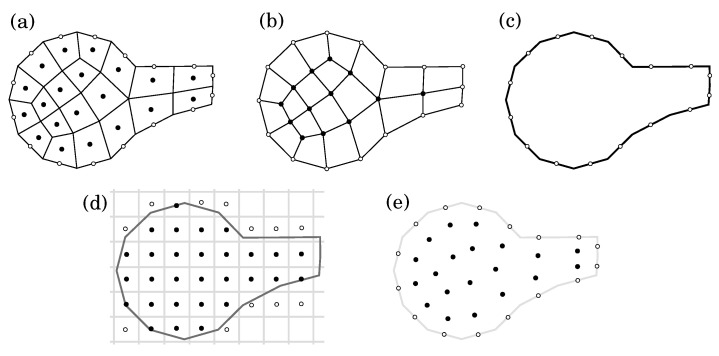
Comparison of space discretizations schemes: (**a**) cell-centered volumes, (**b**) vertex-based volumes, (**c**) boundary elements, (**d**) Cartesian-grid nodes, and (**e**) meshless discretization used in LDD, where black circles depict inner computational nodes, while white circles depict boundary computational nodes.

The research field focusing on simulating viscoelastic flows has now reached a critical juncture marked by a convergence of advances and challenges. Researchers have made significant progress in developing numerical methods, with a significant focus on meshless techniques for modeling the sophisticated dynamics exhibited by viscoelastic materials. The current state of the field is marked by a growing recognition of the importance of accurate simulations for applications ranging from industrial processes like polymer manufacturing to biological systems like blood flow. The initial mathematical models that appeared in the domain of continuum mechanics were defined by Oldroyd [4], yet the Oldroyd-B model continues to be a central focus in research efforts in terms of constitutive models. Upper-convected Maxwell (UCM), Oldroyd-B, and corotational Maxwell models are examples of early constitutive equations that are differential-type equations for the extra stress tensor. Castillo Sánchez et al. [5] critically examined the extensive use of the Oldroyd-B model in predicting instabilities across diverse shearing flows of viscoelastic fluids, emphasizing its qualitative success despite limitations and discussing potential improvements using more realistic constitutive models and open questions in viscoelastic stability. Researchers are eager to investigate its implementation and integration into numerical simulations, recognizing its ability to accurately capture the viscoelastic properties of materials. The exploration and refinement of meshless methods, such as Lagrangian differencing dynamics (LDD) [6], as viable alternatives to traditional mesh-based approaches (see Figure 1), is one notable trend in the current literature. Meshless methods have gained popularity due to their improved ability to handle complex geometries and dynamic fluid interfaces, providing a promising avenue for simulating viscoelastic flows. Despite these advances, obstacles remain. Computational efficiency, stability, and the ability to deal with highly nonlinear viscoelastic behaviors are still being researched. Furthermore, the field is increasingly recognizing the importance of benchmark problems and standardized validation protocols for evaluating the accuracy and reliability of various numerical methods.

The HWNP expresses itself as a lack of simulation stability, resulting in a rapid surge of the numerical solution [1]. Inhibition occurs when the Weissenberg number (or Deborah number) reaches a critical point, which varies due to features involving flow difficulty, spatial discretization, and numerical methods. The degradation of positive definiteness within the conformation tensor, which is an internal variable characterizing the configuration of the polymer chains, is known to be a precursor to the HWNP [7]. In addition, Fattal and Kupferman et al. [7,8] have shown that numerical instabilities occur due to insufficient resolution of spatial stress profiles. This is a concern because viscoelastic flow solutions typically involve stress boundary layers with substantial variations in stress gradients and exponential stress profiles close to geometric singularities. Inadequate representation of stress gradients, e.g., through the use of polynomial interpolations of exponential profiles, results in an underestimation of convective stress fluxes. This error is then compensated for by multiplying the rate of stress growth, ultimately leading to computational errors. The log-conformation representation [9,10,11] not only maintains positive definiteness but also improves the characterization of significant stress gradients by transforming the exponential stress profiles into linear ones. Problems with a high Weissenberg number are crucial in viscoelastic flow because they involve situations where elastic forces have a significant impact on fluid behavior. This leads to the occurrence of complex phenomena like coil-stretch transitions and elastic instabilities, which pose challenges for accurate simulation and practical comprehension in various industrial and scientific applications.

Achieving numerical stability becomes paramount when simulating flows characterized by high Weissenberg numbers, as these regimes are particularly susceptible to error propagation and solution breakdown [5,12]. Verbeeten et al. [13] used the discrete elastic viscous stress splitting technique in combination with the discontinuous Galerkin (DEVSS/DG) method to simulate a polyethylene melt, highlighting the interplay between physical and numerical aspects in achieving stable solutions for complex flows. In order to try to simulate flows with high Weissenberg numbers, Fattal and Kupferman [7,12] suggested the log-conformation formulation in the context of the FDM by utilizing the matrix logarithm of the conformation tensor to improve stability at high Weissenberg numbers. This suggests that logarithmic variables alleviate numerical instability issues, with the remaining challenges focused on accuracy degradation at insufficient resolution. Comminal et al. [1] combined log-conformation formulation with the stream function description for incompressible viscoelastic flows. Fernandes [11] introduced a block-coupled algorithm for computing viscoelastic flows using the log-conformation tensor approach, employing implicit discretization for various terms and validating the algorithm for non-isothermal viscoelastic Oldroyd-B fluid flow. Meburger et al. [14] introduced an approach utilizing the root conformation approach on unstructured meshes, demonstrating stability and validation against experimental data for high Weissenberg numbers and different wall temperatures. Fernandes et al. [15] introduced an improved both-sides diffusion scheme in an FVM framework for simulating viscoelastic fluid flows, demonstrating accurate predictions for benchmark cases and achieving steady-state solutions with refined meshes and a convergence order close to the second order. The work of Pimenta and Alves [9] also enhanced the stability of an FVM viscoelastic solver, demonstrating second-order accuracy in time and space for low Deborah numbers while providing new insights into the vortex dynamics and transient behavior in the 4:1 planar contraction experiment. Giorgi and Morro [16] presented a scheme for modeling viscoelastic materials by introducing constitutive functions based on measures of strain, stress, heat flux, and their time derivatives, ensuring consistency with the second law of thermodynamics and allowing for a wide range of models, including nonlinear thermo-viscoelastic materials with large deformations, encompassing well-known models.

Other studies have investigated turbulence, sediment transport, etc. Brandi et al. [17] investigated laminar-turbulent transition in viscoelastic fluid flows using direct numerical simulation (DNS) and linear stability theory (LST), demonstrating the influence of polymer concentration and elastic forces on critical Reynolds numbers, revealing changes in flow structures for specific parameters. Recent studies also include machine learning (ML) algorithms. For example, the study of Faroughi et al. [18] introduced an ML framework employing random forest, deep neural network, and extreme gradient boosting models to evaluate the coefficient of drag for spherical particles immersed in a viscoelastic fluid, using datasets generated from DNSs.

Due to the memory effects of viscoelastic fluids, the Lagrangian perspective is natural [19]. The Lagrangian perspective is particularly effective in modeling complex flows, where the path and interaction of individual fluid elements are of significant interest, such as in turbulent flows or multi-phase systems. By focusing on the movement and interaction of individual parcels, Lagrangian CFD provides a detailed and dynamic picture of fluid behavior, revealing insights into the underlying physical processes driving flow. Consequently, researchers have investigated extending the most famous meshless method, smoothed particle hydrodynamics (SPH), to viscoelastic flows. Ma et al. [20] presented a two-phase smoothed particle hydrodynamics (SPH) model utilizing the Oldroyd-B constitutive model to predict sediment transport and erosion in free-surface flow under various scouring conditions. Bhattacharya et al. [21] addressed the tensile instability in SPH for weakly compressible fluids by developing an adaptive algorithm using a B-spline function as the SPH kernel, demonstrating effectiveness through dispersion analysis of an Oldroyd B fluid material model and benchmark fluid dynamics simulations. Xu and Yu [22] also addressed the issue of tensile instability in SPH for simulating transient viscoelastic free surface flows, introducing an optimized particle shifting technique and demonstrating its effectiveness through simulations of impacting drops, injection molding, and extrudate swell with comparisons to other numerical methods and techniques. Castelo et al. [23] introduced a moving least squares (MLS) Eulerian meshless interpolation technique, enabling complex mesh configurations while maintaining overall accuracy, demonstrated through simulations of generalized Newtonian and viscoelastic fluid flows.

The following study addresses the need for precise and efficient numerics to capture the complex dynamics of viscoelastic materials in a wide range of applications, from industrial processes to biological systems. The primary goal of this work is to extend the meshless Lagrangian differencing dynamics (LDD) method [6,24,25,26] to simulate viscoelastic flows by incorporating the Oldroyd-B constitutive model to achieve accurate and efficient modeling of complex viscoelastic fluid behaviors. LDD is a numerical method that combines Lagrangian and mesh-free approaches to capture the dynamics of these fluids. In the context of viscoelastic simulations, meshless Lagrangian discretization offers a distinct advantage due to its inherent property tracking by particles, facilitating the incorporation of memory effects. This study focuses on derivative-based viscoelastic models, although integral formulations offer a viable alternative approach. The LDD method does not suffer from tensile instability problems and can handle large negative pressures Bašić et al. [24], as compared to SPH schemes, which require numerical remedies [21,22,27,28,29]. It will be shown how the method proposed in this paper provides a distinct advantage by avoiding the limitations of traditional mesh-based approaches. Its meshless Lagrangian nature allows for a more accurate representation of complex geometries and dynamic fluid interfaces, with easier avoiding of the HWNP. The significance of this study lies in its potential to improve simulation accuracy, thereby contributing to advancements in fields as diverse as polymer processing, biomedical engineering, and materials science. The following benchmarks are used to assess LDD’s efficacy in simulating such flows: a lid-driven cavity, the three-dimensional impact of a droplet, sudden contraction in a 4:1 channel, and extrusion die swelling. The applicability of the method to real polymer fluid response is demonstrated through the successful simulation of benchmarks, which are relevant to industrial polymer processing scenarios.

The rest of the paper is organized as follows. Section 2 presents the fundamental governing equations that form the mathematical backbone of the study. In Section 3, the numerical procedure employed to solve these equations is outlined, detailing the computational methods and algorithms utilized. In Section 4, a comprehensive analysis of the results is offered, coupled with a thorough discussion that interprets the findings and explores their implications. Finally, Section 5 concludes the paper, summarizing the key outcomes of the research and suggesting potential directions for future work.

## 2. Governing Equations

Generalized Navier–Stokes equations (NSE) in strong vector form [25] are used to describe the flow of an incompressible fluid that exhibits viscoelastic effects. The conservation of the momentum and mass and the Lagrangian advection can be written, respectively, as follows:

(1) $$\frac{Du}{Dt}=\frac{1}{\rho }\left\{-\nabla p+\nabla \cdot \tau_{s}+\nabla \cdot \tau_{p}\right\}+a_{ext},$$

(2) ∇·u=0,

(3) $$\frac{Dx}{Dt}=u,$$

where ρ is the constant fluid density, u is the velocity vector, *p* is the pressure, x is the position of the Lagrangian parcel, $a_{ext}$ is the external acceleration vector field, $\tau_{s}$ is the solvent diffusion stress tensor, and $\tau_{p}$ is the polymeric extra-stress tensor. The above equations are dependent on the time, t≥0, and space defined in the fluid domain Ω, even though the equations do not specify it explicitly. In the Lagrangian context, the momentum Equation (1) describes the acceleration of a fluid parcel. The fluid parcel is moving along its streamline, i.e., according to the velocity field based on Equation (3), while the movement remains incompressible due to the continuity constraint (2). The momentum Equation (1) splits the stress tensor τ into the viscous-solvent contribution, $\tau_{s}$, and the (viscoelastic) polymer stress contribution, $\tau_{p}$. Consequently, the total dynamic viscosity of the fluid is also split into the solvent and polymeric contributions, i.e., $\mu =\mu_{s}+\mu_{p}$. In the following subsections, the solvent and polymeric terms introduced in Equation (1) are described and explained.

### 2.1. Solvent Viscous Stress

The viscous stresses that stem from the solvent diffusion process are represented using a symmetric tensor:

$$\tau_{s}=2\mu_{s}E,$$

where E is the symmetric tensor denoting the rate of deformation:

E=12∇u+∇u⊤.

The diffusion–acceleration term present in Equation (1) equals the divergence of the tensor, $\nabla \cdot \tau_{s}/\rho$. Therefore, for constant viscosity fluids, μ= const., the diffusion acceleration depends on the Laplacian of the velocity:

(4) $$\nabla \cdot \tau_{s}=\mu_{s}\nabla^{2}u,$$

while for variable-viscosity fluids, i.e., for $\mu =f\left(x,u,p,\ldots \right)$, diffusion acceleration must take the gradients of viscosity and velocity into account [30]:

(5) $$\begin{array}{cc} \nabla \cdot \tau_{s} & =\nabla \cdot \left(\mu_{s}\nabla u\right)+{\left(\nabla u\right)}^{⊤}\nabla \mu_{s}. \end{array}$$

Flowing materials that are devoid of elasticity effects can be effectively modeled using the diffusion term with variable viscosity without including $\tau_{p}$ because their nonlinear viscosity characteristics primarily respond to shear rates without memory effects or elastic recovery, i.e., it approximately holds $\mu =f\left(E\right)$. In the proposed numerical method, the viscosity function can be described using any non-Newtonian model, some of which were validated in the fully Lagrangian context using the LDD method [25,26].

### 2.2. Polymeric Stress

Viscoelastic models exhibiting both viscous and elastic properties require the incorporation of an extra stress tensor $\tau_{p}$ into Equation (1), which is essential for capturing the fluid’s memory effects and its ability to store and release elastic energy. The extra stress tensor captures the exchange between the fluid’s viscous dissipation and elastic response under varying flow conditions based on the current state of deformation and deformation history. The evolution of the polymeric viscoelastic stress is defined by the upper-convected derivative:

(6) $${\overset{\nabla }{\tau }}_{p}=\frac{D\tau_{p}}{Dt}-\tau_{p}\cdot \nabla u-{\left(\nabla u\right)}^{T}\cdot \tau_{p},$$

where the symmetric assumption of the extra stress tensor can be used to simplify the following equation:

(7) $${\overset{\nabla }{\tau }}_{p}=\frac{D\tau_{p}}{Dt}-2\tau_{p}\cdot E,$$

where the dot-product between two tensors may be defined using the matrix product, $\tau_{p}\cdot E=E\tau_{p}$. Within viscoelastic constitutive models, upper or lower convected time derivatives are employed to guarantee frame invariance in the transport of tensorial attributes [31]. In this study, the Oldroyd-B model is employed for the polymeric component of the fluid to consider the fluid’s instantaneous elastic response and its rate-dependent viscous behavior, as a demonstrative example to validate the proposed methodology. The Oldroyd-B fluid presents one of the simplest constitutive models capable of describing the viscoelastic behavior of dilute polymeric solutions under general flow conditions. It provides linear viscoelastic modeling of dilute polymer solutions based on the microscopic dumbbell with a linear entropic spring [32]. The viscoelastic stress is governed by the Oldroyd-B constitutive equation:

(8) $$\tau_{p}+\lambda_{1}{\overset{\nabla }{\tau }}_{p}=2\mu_{p}E,$$

where $\lambda_{1}$ is the relaxation time over which the elastic stress relaxes when the strain is constant. Some other models were also developed by Rivlin, Green, Tobolsky, Ericksen, Lodge, Phan-Thien, Tanner, and Giesekus et al. (please see [31] for the references). In a truly Lagrangian framework, which inherently tracks the history and evolution of stress in a specific material element moving with the flow, Equation (8) is an ordinary differential equation (ODE). Therefore, Equation (8) may be resolved in time by any numerical integration method.

The Oldroyd-B model represents the limiting scenario of linear viscoelasticity for the majority of non-linear models. Due to dissipative constitutive instability, it is known to produce unreliable projections in purely extensional deformation. Nevertheless, due to its increased susceptibility to numerical instabilities compared to the more reliable non-linear viscoelastic models, it serves as a valuable model for evaluating the performance of the numerical solver. For real polymer materials, $\lambda_{1}$ is associated with the time it takes for polymer chains to relax or reconfigure after the application of stress.

The inclusion of nonlinearity in the constitutive models enhances the stability of the flow and improves the convergence of the numerical solvers [33]. Another parameter relevant to the Oldroyd-B model that is not evident in Equation (8) is the retardation time $\lambda_{2}$, which relates the decay of memory effects in the material to the solvent diffusion effects. $\lambda_{2}$ is related to the delay in the elastic response of the polymer solution. Through the viscosity-ratio connection, $\beta =\mu_{s}/\mu =\lambda_{2}/\lambda_{1}$, the simulations pre-process polymer and solvent viscosities. If the retardation time is negligible, $\lambda_{2}=0$, then the formulation reduces to the UCM model, which eliminates the contribution of the solvent viscosity.

## 3. Numerical Procedure

### 3.1. Lagrangian Splitting Scheme

Numerical methods that separate velocity and pressure lead to reduced systems of equations with fewer interconnected variables and prevent saddle-point problems. Hence, such methodologies are attractive for certain large-scale problems. Therefore, these methodologies are appealing when dealing with particular large-scale problems. This paper presents a numerical method for solving the pressure Poisson equation (PPE) (re)formulation of the Navier–Stokes equations for viscoelastic fluids. The approach is specifically designed for fully Lagrangian scenarios. The split scheme that describes the pressure–velocity–advection steps may be solved using any Lagrangian method that introduces consistent spatial operators. In this work, LDD [6] is employed due to its validation of viscoplastic non-Newtonian flows [25,26], its ability to reach second-order accuracy [34], and its performance and extensibility [24]. The proposed numerical procedure does not correct the HWNP using the log-conformation or any other numerical approach. This is carried out to test the limits of the fundamentals of the proposed numerical scheme, while stabilization approaches may be added in future work.

A key aspect of establishing a consistent numerical scheme, which decouples the velocity and pressure, is the determination of the proper pressure equation [35,36]. The pressure equation is employed as a substitute for the continuity constraint (2), i.e., the requirement that the velocity field always remains solenoidal. The pressure field, $p\left(x\right)$, should yield the acceleration of fluid particles through the pressure gradient ∇p, such that Equation (2) is satisfied at all discrete time instants.

The equation of the pressure field to be solved is a scalar Poisson equation, which is derived by applying the divergence operator ∇· onto the momentum Equation (1) [6]. Furthermore, the boundary condition at solid walls is obtained by dotting the momentum Equation (1) by the wall-normal n [37]. On the free surface, the atmospheric pressure is imposed through the Dirichlet boundary condition. These operations yield the following system of equations:

(9) $$\nabla^{2}p=\nabla \cdot \left\{-\rho \frac{Du}{Dt}+\rho \;a_{\mathrm{ext}}+\nabla \cdot \tau_{s}+\nabla \cdot \tau_{p}\right\},\;x\in \Omega ,$$

(10) $$\frac{\partial p}{\partial n}=n\cdot \left\{-\rho \;\frac{DU}{Dt}+\rho \;a_{\mathrm{ext}}+\mu \nabla^{2}u\right\},\;x\in \Gamma_{wall},$$

(11) $$p=0,\;x\in \Gamma_{fs},$$

where all the introduced terms are described as follows. For the first term in Equation (9), the divergence of the Lagrangian acceleration is numerically defined as follows:

(12) $$\langle \nabla \cdot \frac{Du}{Dt}\rangle =\nabla \cdot u\frac{\left|u\right|}{\Delta },$$

where 〈〉 are the brackets denoting numerical approximation. Incorporating the numerically accumulated divergence of the velocity field, ∇·u, into Equation (9) is important to damp the compressibility in time [36]. Therefore, it is often referred to as the ‘divergence damping term’ [38]. While it is unclear how to use it and interpret it in the Eulerian context [35,38,39,40], it has a physical meaning in the Lagrangian context. The right-hand side of Equation (12) can be interpreted as ‘how fast the divergence propagates to neighboring Lagrangian parcels’, which is damped within the time step.

In this study, the gravity vector field is taken as constant; therefore, the second term in the right-hand sides of Equations (9) and (10) may be neglected, i.e., $\nabla \cdot a_{ext}=0$, while for variable gravity fields, it should be taken into account.

The third term is the Laplacian term, which accounts for the viscous velocity–divergence (please see Appendix A):

(13) $$\langle \nabla \cdot \left(\nabla \cdot \tau_{s}\right)\rangle =\nabla \cdot \left[\mu_{s}\nabla \left(\nabla \cdot u\right)\right].$$

As the first term approximated by Equation (12) accounted for accumulated overall divergence, the last term accounts for the current stress caused by elasticity (obtained by solving Equation (8)):

(14) $$\langle \nabla \cdot \left(\nabla \cdot \tau_{p}\right)\rangle =\nabla^{2}\mathrm{tr}\left(\tau_{p}\right),$$

where ‘the Laplacian of the polymeric tensor–trace’ is taken as the appropriate approximation. The derivation of Equation (14) is explained in Appendix B and backed by observations by Kumar et al. [41]. Note that, in Equation (10), for simplicity, we take μ= const. at $\Gamma_{\mathrm{wall}}$, even for variable-viscosity fluids. This is a sufficient approximation for second-order methods since the viscosity is already extrapolated from fluid to wall (and second extrapolation would not yield better physics).

The linear system is constructed based on Equations (9)–(11). The terms of the right-hand side of the system were explained in the text above, while the left-hand side discretization of the spatial operators (Laplacian and directional derivative) are explained in the next section. Detailed information on the solvability and convergence of the system of Equations (9) and (10) is given in [6,24]. In this study, the linear system is solved using the right-preconditioned BiCGStab.

After solving the PPE defined above, the pressure field may be included in the momentum Equation (1) to solve it for the velocity. Furthermore, the obtained velocity field is used to solve the extra stress Equation (8) and the advection Equation (3). The equivalence of the so-called PPE reformulation of the NSE has been widely analyzed [35,36,37,38,42]. Compared to [43], the PPE reformulation of the NSE in this paper is carried out in the Lagrangian context by replacing the convective operator issues and time-step limitations with the advantages of Lagrangian advection. The discretization and solution of the decoupled pressure–velocity–extra-stress–advection system are explained in the following subsections.

### 3.2. Spatial Discretization

In the LDD method, the computational domain is discretized as a point cloud, thus eliminating the need for topological information. In discrete equations, a Lagrangian particle *i* defines the central node, while j∈N defines its neighbor node from the set neighboring nodes, N. For simplicity of presenting the following equations, the subscript ij shall define the difference between values of two neighboring nodes, ${□}_{ij}={□}_{j}-{□}_{i}$. Particle *i*’s neighbors, j∈N, are searched around the location $x_{i}$ to be within the compact radius *h*, i.e., $0<\left|x_{ij}\right|\leq h,\;\forall j\in N$. For the incompressible flow, the closest neighbors around $x_{i}$ are organized to be distanced close to some initial particle spacing, Δ, and the first ring of neighbors around the particle *i* is taken for the discretization of spatial operators [6]. Therefore, the recommended values of the compact radius lie within the interval 1.3Δ<h<2.5Δ. In this work, h=1.7Δ is taken, which is on the safe side to capture the full ring of closest neighbors. The weighting function describes how much a neighbor contributes to the interaction between nodes based on the distance $x_{ij}$ between the two neighbors [34]. In this work, the Wendland kernel is used as the weighting function, $W\left(\left|x_{ij}\right|\right)$.

The term Lagrangian differencing was introduced to emphasize that the numerical method is built upon finite differences in the Lagrangian context. Consequently, the Navier–Stokes equations are solved in strong form, i.e., the equations are directly discretized by substituting the discrete LDD spatial operators. The discrete approximations of the continuous spatial derivatives are based on the renormalization tensor and the offset vector:

(15) $$B_{i}={\left(\sum_{j\in N}W_{ij}\;x_{ij}\;x_{ij}^{⊤}\right)}^{-1},$$

(16) $$o_{i}=\sum_{j\in N}W_{ij}\;x_{ij},$$

which are calculated at each time-step for each point’s neighborhood. The first-order derivatives discretized in the LDD context are defined as follows:

(17) $${\langle \nabla f\rangle }_{i}=\sum_{j\in N}W_{ij}\;B_{i}x_{ij}\;f_{ij},$$

(18) $${\langle \nabla f\rangle }_{i}=\sum_{j\in N}W_{ij}\;B_{i}x_{ij}\;f_{ij}^{⊤},$$

(19) $${\langle \nabla \cdot f\rangle }_{i}=\sum_{j\in N}W_{ij}\;B_{i}x_{ij}\cdot f_{ij},$$

(20) $${\langle \nabla \cdot F\rangle }_{i}=\sum_{j\in N}F_{ij}\;\left(W_{ij}B_{i}x_{ij}\right),$$

where *f* is a scalar field, f is a vector field, and F is a tensor field. It is noticeable that the term $W_{ij}B_{i}x_{ij}$ may be understood as a component of the Hamilton derivative operator [6]. The term ’component’ is used because the final outcome is achieved by adding together individual neighboring weights, ∀j∈N. The only second-order derivative needed to solve the NSE using the proposed numerical scheme is the Laplacian, which is approximated in the LDD context:

(21) $${\langle \nabla \cdot \left(\phi \nabla f\right)\rangle }_{i}=d\;\frac{\sum_{j\in N}\;L_{ij}\left(\phi_{i}+\phi_{j}\right)f_{ij}}{\sum_{j\in N}\;L_{ij}{\left|x_{ij}\right|}^{2}},$$

where $L_{ij}\equiv W_{ij}\left(1-x_{ij}\cdot B_{i}o_{i}\right)$, and ϕ are scalar fields. Equation (21) also has the same formulation for a vector function, f. Thorough analysis of the LDD spatial operators may be found in [34].

Since the fundamentals of spatial discretization are introduced through LDD operators, the splitting scheme may be possible.

### 3.3. Temporal Discretization

To solve the NSE in time using the proposed method, three subproblems that rely on Lagrangian derivative, D/Dt, are approximated:

- Equation (1) is used to obtain the velocity field based on the discretization of Du/Dt;
- Equation (3) is used to advect Lagrangian nodes based on the discretization of Dx/Dt;
- Equation (8) is used to calculate the extra-stress tensor based on the discretization of $D\tau_{p}/Dt$.

The variable time-step size during the numerical simulation is chosen based on the Lagrangian CFL (LCFL) constraint [44], which is defined as follows:

(22) $$\mathrm{LCFL}=\delta t{\left|\nabla u\right|}_{\infty }.$$

The condition restricts the time-step size based on the maximum strain in the flow, i.e., the *L*-infinity norm ${\left|\nabla u\right|}_{\infty }$, which is independent of the discretization size, as compared to classical Eulerian CFL conditions. Therefore, the Lagrangian constraint for the time-step size is usually more relaxed than its Eulerian counterpart, as the constraint LCFL<1 guarantees that characteristic curves do not intersect during a time-step.

#### 3.3.1. Momentum Equation

The backward differencing formula of the second order (BDF2) used in the context of variable time-step sizes is employed to discretize the Lagrangian derivative for the velocity:

(23) $$\langle \frac{Du}{Dt}\rangle =\frac{1}{\delta t}\left[\left(\frac{1+2r_{t}}{1+r_{t}}\right)u_{n+1}-\left(1+r_{t}\right)u_{n}+\left(\frac{r_{t}^{2}}{1+r_{t}}\right)u_{n-1}\right],$$

where $r_{t}$ is the ratio of the current and previous time-step sizes, $r_{t}=t_{n}/t_{n-1}$. Solving $u_{n+1}$ by substituting Equation (23) in the momentum Equation (1) is carried out by leaving the velocity $u_{n+1}$ and the diffusion terms on the left-hand side while putting everything else (pressure gradient and viscoelasticity divergence) on the right-hand side. The momentum Equation (1) incorporates the pressure field as an explicit term, which is obtained by solving the system of Equations (9) and (10). It should be pointed out that the scheme handles constant and variable viscosity fluids. For variable-viscosity fluids, the viscosity can depend on many parameters, $\mu =f\left(x,u,p,\ldots \right)$. During each time-step, before solving for the pressure and velocity fields, the variable viscosity scalar field may be calculated [25,26].

#### 3.3.2. Advection

In Lagrangian CFD methods, the fluid dynamics are analyzed from a particle-centric perspective. This approach involves tracking discrete fluid parcels as they advect and carry their properties. The trajectory of each parcel is computed by integrating its velocity over time, allowing the simulation to capture the evolving flow patterns and interactions within the fluid. The Lagrangian particle movement is defined by Equation (3), while the first order derivative in time describes the change in the particle location within the time-step:

(24) $$\langle \frac{Dx}{Dt}\rangle =\frac{1}{\delta t}\left(x_{n+1}-x_{n}\right).$$

Therefore, by substituting Equation (24) into Equation (3), the new location is solved as follows:

(25) $$x_{n+1}=x_{n}+u_{n+1}\delta t.$$

The numerical particle is explicitly moved in time (advected) using the newly obtained velocity $u_{n+1}$. In the discrete context, advection always causes compressible results [45] due to the order (truncation) of the method, imperfect convergence and floating point errors, or simply because neighboring streamlines will collide or separate for a large discrete value of δt. Due to these inherent properties of Lagrangian advection, all particles are reordered after advection [6] using an iterative algorithm [46]. In summary, the semi-implicit scheme for advection is established by combining the explicit movement and implicit reordering.

#### 3.3.3. Extra-Stress Evolution

In the case of viscoelastic flows, the viscoelasticity tensor is evolved in time by integrating Equation (8). Since the particles are carrying tensorial properties along their trajectories, the tensorial Lagrangian derivative $D\tau_{p}/Dt$ is discretized using the first-order derivative:

(26) $$\frac{D\tau_{p}}{Dt}=\frac{1}{\delta t}\left(\tau_{p,n+1}-\tau_{p,n}\right),$$

and Equation (26) is substituted into Equation (8). By leaving only $\tau_{p,n+1}$ on the LHS, the viscoelastic tensor is explicitly evaluated at each time step.

### 3.4. Final Algorithm

A single time step contains the following sub-steps:

1. Advect particles and reorder their positions, as described in Section 3.3.2.
2. Generate boundary particles on walls by projecting closest fluid particles into walls, as described in [6].
3. Prepare spatial operator information, i.e., (15) and (16).
4. Solve the pressure equation, as described in Section 3.1.
5. Solve for velocity, as described in Section 3.3.1.
6. Calculate the viscoelasticity tensor, as described in Section 3.3.3.
7. Calculate the next allowable time-step size according to (22).

In the discrete Lagrangian context, the split-step scheme is characterized by its decoupled pressure–velocity–advection steps, which are resolved once within each time increment. This method, inherently non-iterative in nature, represents an optimal approach performance-wise for the resolution of NSE [6,35,40]. However, it should be acknowledged that the scheme cannot mandate absolute convergence due to the absence of multiple iterations per time-step (along with other errors, like truncation order, etc.). Consequently, it is necessary to regulate time-stepping to ensure global convergence and maintain the stability of the system in a discrete context. In conclusion, divergence in the velocity field is always damped in time, while the time-step size controlled using Equation (22) controls that there is no divergence amplification and numerical blow-up.

## 4. Results and Discussion

### 4.1. Lid-Driven Cavity

The lid-driven cavity benchmark is important for evaluating viscoelastic flows to exhibit intricate flow behaviors such as non-linear velocity profiles, vortical structures, and the formation of elastic instabilities [25,47,48,49]. The lid-driven cavity setup creates a confined space in which these complex flow patterns can manifest, allowing for a thorough assessment of how well a simulation method captures these behaviors. Viscoelastic fluids frequently exhibit shear-thinning behavior, in which viscosity decreases as the shear rate increases. Variable shear rates across the cavity in the lid-driven cavity allow evaluation of how accurately a simulation method represents this non-Newtonian behavior. The benchmark calls for the creation of recirculation zones in which the flow reverses direction. Because of their elasticity, viscoelastic fluids can have altered or intensified recirculation. Accurately predicting the size, shape, and behavior of these zones is critical in assessing the fidelity of a simulation method in capturing viscoelastic effects. Despite its simple geometry, this problem is regarded as exceptionally challenging to solve, particularly at large Weissenberg numbers, since the flow exhibits specific characteristics that combine shear and extensional deformations.

The LDD was used to solve a benchmark test for lid-driven cavity flow. A square geometry representing the cavity, with a unit length *L* = 1, is filled with fluid. The density of the fluid is set to ρ=1, and the maximum velocity of the lid is U=1. A relatively high Weissenberg number for the simulation [50] is chosen as Wi=λU/L=3, while a constant retardation ratio is applied β=12. By setting the Reynolds number to a negligible value, $Re=\rho UL/\mu =5\cdot 10^{-4}$, the flow is conceptualized as a creeping flow. Near the sliding lid, the extensional rate reaches high values [51]. Because the cavity is a closed system without inlets and outlets, recirculating material accumulates the extra stress quicker than it relaxes [1]. The flow may not achieve a steady-state solution and, thus, show elastic instabilities. Numerically, the modeling of the viscoelastic lid-driven cavity flow at high Weissenberg numbers is as difficult as its Newtonian equivalent at high Reynolds numbers [48,51]. The corner singularities may be treated by a controlled amount of leakage [48] or by modifying the lid velocity profile [1]. Similar to [1], in this study, regularization is applied to the tangential velocity profile of the lid to eliminate the stress singularity at the corners. The profile of the lid’s tangential velocity is imposed as follows:

$$u_{x}\left(x,t\right)=16x^{2}{\left(1-x\right)}^{2}\left[0.5+\tanh\left(8\left(t-0.5\right)\right)/2\right]U,\;x\;\epsilon \left[0,1\right],$$

where the hyperbolic tangent smooths the acceleration of the lid while the tangential velocity is scaled exponentially away from corners. By doing so, the stagnation points situated at the corners remain unaffected by any deformation rate, thereby deflecting the formation of stress gradients that have the potential to increase exponentially.

**Figure 2:**
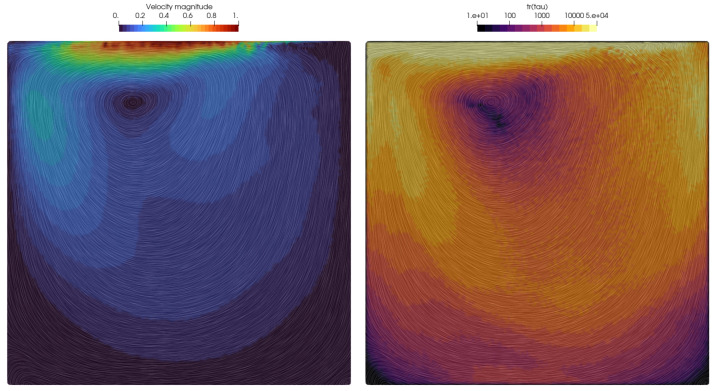
Contour plot of the instantaneous velocity magnitude with streamlines (**left image**) and the trace of the extra stress tensor (**right image**) for the lid–driven cavity flow simulation, Wi=3 and $Re=5\cdot 10^{-4}$.

**Figure 3:**
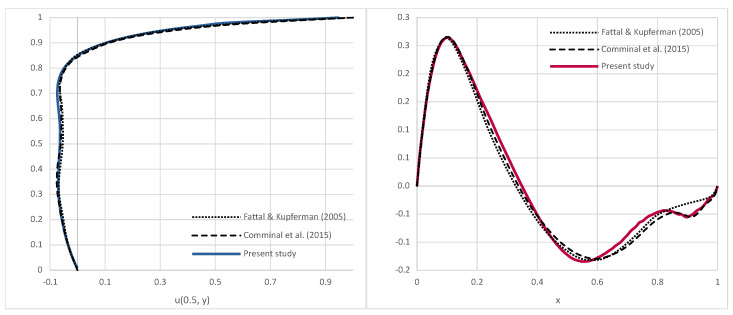
The horizontal velocity profile along x=0.5 and vertical velocity profile along y=0.75 for the lid–driven cavity flow simulation, Wi=3 and $Re=5\cdot 10^{-4}$ [1,12].

To maintain similarity with discretizations of referenced results [1,12,50], the initial point cloud was generated as a 100 × 100 grid, i.e., the initial point spacing was Δ=0.01. The results provided in Figure 2 are compared to the works of [50] and [1], which introduced a stream function based on log-conformation formulation [12] for incompressible viscoelastic flows to ensure the positive definiteness of the conformation tensor, addressing high Weissenberg number challenges. The simulation results successfully captured the upstream shift of the primary re-circulation vortex, i.e., viscoelasticity, which has broken the symmetry that is seen in experiments with the Newtonian creeping flow. The smaller corner eddies were also induced at the bottom of the cavity. In reality, at the bottom corners, there is an infinite series of vortices, diminishing in size and strength as the corner approaches [48]. By comparing the velocity magnitude results shown in Figure 2 to those from [50], it may be seen that the non-symmetry of the velocity in the cavity is well predicted. A characteristic feature of this benchmark is the upstream deformation of streamlines in the upper-right corner, i.e., curve-shaped streamlines due to the significant gradient of the extra stress shown on the right image in Figure 2. This area becomes unstable quickly if the tangential velocity profile of the lid is not regularized. Consequently, the velocity magnitude near the lid experiences larger gradients near the downstream corner compared to the upstream area. The accuracy of the vortex center and obtained velocity field may be seen in Figure 3, where the simulated velocity profiles are given for a horizontal and vertical section and compared to those from [1,12]. The results obtained by the LDD method show the bump in the vertical velocity profile near the right wall, which was also obtained in [1] but not in [1]. This bump in velocity magnitude may also be seen from the contours of the velocity magnitude due to the rise in the extra stress, which is rendered in Figure 2. The peaks and overall shape of the graphs are well aligned. The LDD solution exhibits slightly steeper gradients in the vertical velocity profile, which will be investigated in the future.

### 4.2. Droplet Impact

This section examines the effects of a three-dimensional viscoelastic fluid droplet colliding with a rigid plate. Understanding droplet impact dynamics is important in many fields, including inkjet printing, spray coating, and biomedical applications such as drug delivery. Droplet impact involves intricate interactions between a viscoelastic fluid and a solid surface. When hit, viscoelastic fluids exhibit deformation, stretching, and breakup, making it difficult for simulation methods to model the fluid’s response to external forces accurately. The simulation is initialized by a fluid droplet described as a sphere with diameter of $D_{0}=20$ mm, positioned 40 mm above the rigid plate. The droplet has initial downward velocity $V_{0}=1$ m/s and is allowed to fall under the Earth’s gravity conditions g=9.81 m/s^2^, resulting in the Froude number at impact Fn=2.26. The density of the fluid is ρ=1000 kg/m^3^, and the Oldroyd-B parameters are chosen as μ=4 Pa·s, $\lambda_{1}=0.02$ s, and $\lambda_{2}=0.002$ s. This results in Re=5, β=0.1, and Wi=1. The relaxation time dictates the elastic response of the droplet during the impact, and the viscosity dictates diffusion of advection and elasticity, as well as its final shape.

**Figure 4:**
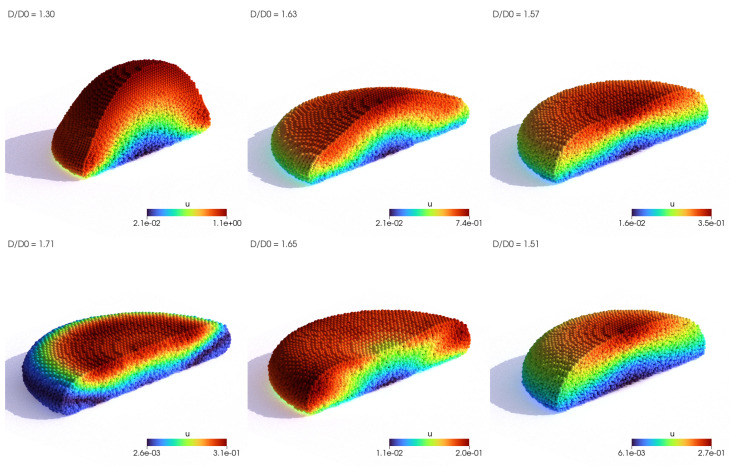
Evolution of droplet deformation during impact, rendered with the velocity magnitude. The simulation with point spacing Δ=0.4 mm is rendered to show the solver capability to reproduce viscoelasticity effects for coarser point-cloud resolutions.

**Figure 5:**
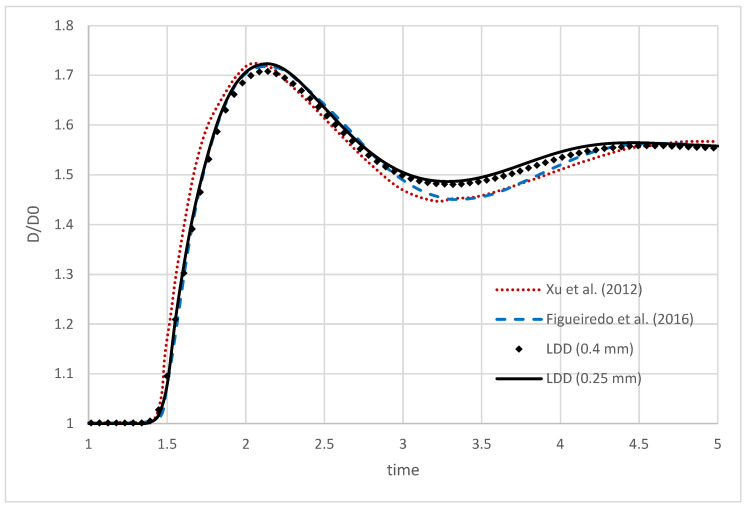
Comparison of the simulated evolution of droplet diameter during the impact [29,52].

Two resolutions of the droplet were tested with initial point spacing of Δ=0.4 mm and Δ=0.25 mm, which led to 60,000 and 250,000 fluid Lagrangian points, respectively. The evolution of the coarser droplet simulation is visualized in Figure 4, in which the impacting droplet is sliced in half to analyze the viscoelastic effects. The magnitude of the velocity field has the potential to explain the evolution of the droplet shape. Upon impact, the droplet undergoes vertical compression and horizontal elongation (seen in the first three images in Figure 4). In contrast to Newtonian flows, at the maximum compression and horizontal expansion of the droplet, the volume is distributed around the edges of the droplet. The transition of the energy from the center of the droplet can be seen in the fourth and fifth images in Figure 4. Eventually, the elongated shape of the droplet compresses back to the final oval shape. The evolution of the droplet diameter is thus characterized by rapid fluctuation, alternating between the local maximum and minimum peak until it eventually settles on an intermediate value. This is shown in Figure 5, and the results of two simulations conducted using the proposed method are compared to numerical results obtained by Xu et al. [29] and Figueiredo et al. [52]. The results obtained from this study align well with the existing findings from [29,52], providing additional confirmation of the accuracy of the suggested methodology. The slope and the peak of the diameter elongation are almost identical to the results in [52]. Nevertheless, one inconsistency is that the local minimum peak in the simulations is not as profound as the ones noticed in the references. The discrepancy is likely attributed to the imposition of the no-slip boundary condition for sliding free-surface points along the plate, hindering the droplet’s compression after elongation. Further investigation is needed to confirm this hypothesis. Compared to Xu et al. [29] and other SPH methods, no tensile instabilities and fracturing phenomenons were detected.

### 4.3. 4:1 Planar Contraction

**Figure 6:**
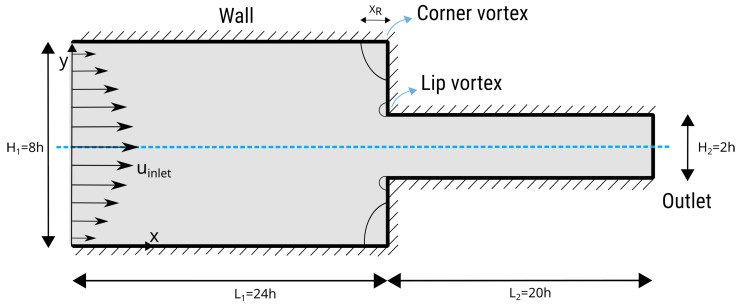
The geometry of the 4:1 planar contraction and the positions of the recirculation vortices are shown. XR is the corner vortex’s reattachment length.

The ‘4:1 planar contraction’ problem is an essential benchmark test for viscoelastic flow solvers in computational fluid dynamics, particularly when dealing with viscoelastic fluid dynamics. This configuration show in Figure 6, which involves a sudden contraction of the flow channel from wider to narrower, creates a complex flow situation useful in polymer processing and inkjet printing [53]. This benchmark is important because it clarifies and tests the solver’s ability to accurately capture viscoelastic fluids’ complex behavior under sudden geometric changes. Extrusion is a common polymer manufacturing method for elongated, consistent components. A die liquefies and shapes plastic to the desired cross-sectional shape. Due to flow pattern reorganization, material forced out through the die expands at exit. The flow transitions from parabolic profiles within the die, constrained by the walls, to a uniform profile outside the die, where the free surfaces are in equilibrium, which is termed ‘extrudate swelling’. Swelling can cause distortions in the extrudates, leading to the development of non-axisymmetric profiles. Flow perturbations hinder polymer extrusion efficiency when extrusion velocities are high. This benchmark test also tests the solver’s numerical stability and robustness since steep velocity gradients and high strain rates in the contraction zone can cause convergence or numerical diffusion issues.

**Figure 7:**
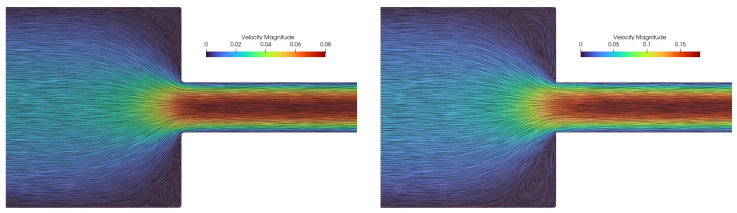
Contour plot of the velocity magnitude and streamlines for the 4:1 planar contraction for Wi=5 (**left image**) and Wi=10 (**right image**).

The results of the velocity field for two simulations, Wi=5 and Wi=10, are shown in Figure 7. For the simulated case, Wi=5, a complex pattern that consists of two merged recirculation zones is visible. The smaller vortex recirculates near the contraction corner, while the larger vortex stretches from the smaller vortex to the adjacent wall. The post-processing artifacts that are visible around the recirculation corner in Figure 7 are due to the Lagrangian nature of the flow, i.e., it is hard to capture streamlines of mesh-free points that are advecting with near-zero velocity. The corner-vortex length for the steady solution for Wi=5 is measured to be around $X_{R}=1.2h$, while for Wi=5, the length is measured as $X_{R}=2.3h$. The corner-vortex lengths correspond to the values reported by Moreno et al. [54] and Afonso et al. [55]. Wittschieber et al. [56] and Niethammer et al. [57] present simulations in which the size of the lip vortex continuously increases as the Weissenberg number increases while the corner vortex decreases in size. They also note that the results are highly dependent on the mesh resolution. In the case of LDD, we have not found that the shapes of lip and corner vortices significantly change due to different point-cloud resolutions. They decrease in size for increasing Weissenberg numbers up to Wi≈3. Afterwards, the vortex increases as the Weissenberg number increases. The change between the two regimes is located at smaller Weissenberg numbers when a coarser mesh is used [57]. For example, in [57], the corner vortex starts to abruptly spread for Wi>10, while in [54,55], the corner vortex starts to abruptly spread for Wi>4. All references agree that the stress cannot be perfectly resolved in the small region near the re-entrant corner, which is not surprising because the corner singularity exists regardless of the computational grid resolution. Since the current implementation of the LDD method does not include adaptive refinement of the Lagrangian point cloud that is important in the boundary layer region, in this study, the lip vortex for small Weissenberg numbers Wi<3 could not be analyzed. Therefore, Figure 7 presents the case of Wi=5, in which the lip vortex has a significant size compared to the point-cloud resolution, i.e., spacing between neighboring points. From the figure, it is also evident that the simulation with a larger Weissenberg number produced larger corner vortices.

**Figure 8:**
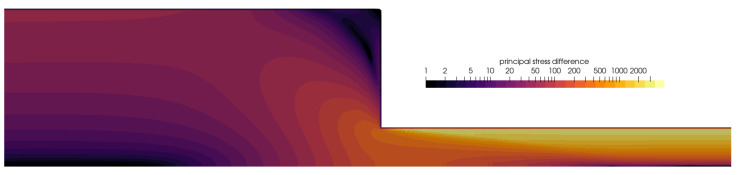
Contours of the principal stress difference (PSD) around the 4:1 planar contraction, simulated for Wi=5.

Figure 8 renders the principal stress difference (PSD) around the contraction for Wi=5, which is calculated based on the components of the extra stress tensor as follows:

$$PSD=\sqrt{{\left(\tau_{xx}-\tau_{yy}\right)}^{2}+4\tau_{xy}^{2}}.$$

The contour plot with the logarithm scale of the PSD is discretized in 24 levels so that relevant transition of values may be analyzed and compared to experimental data by Wang and Wang [53] and Verbeeten et al. [13]. The numerical results show that the predicted PSD pattern agrees well with the experimental data measured by the flow-induced birefringence (FIB) device. The most important details, i.e., the pattern inside the vortex and the “butterfly pattern” of PSD [53], are well-predicted. In other words, the contours in the vortex are correctly stretching, while the pattern around the sharp corner symmetrically spreads upstream and contracts downstream.

**Figure 9:**
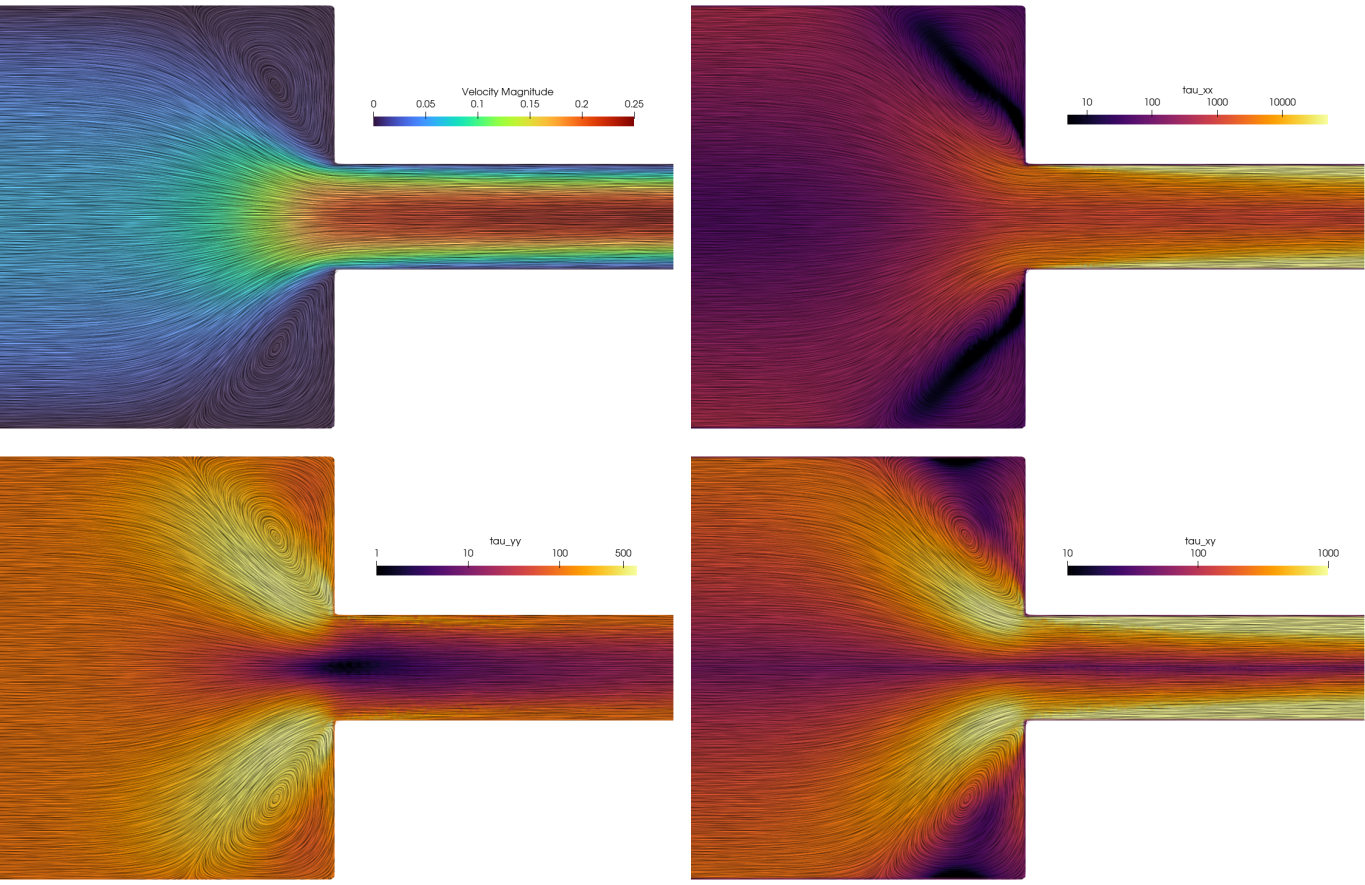
Contours of the velocity magnitude (**upper left image**) and extra stress tensor components for the 4:1 planar contraction simulation for Wi=14 and Re=0.01.

Since the standard Oldroyd-B formulation is used in this work, instead of log-conformation methods that are usually classified as numerically more stable [11,54], a simulation with parameters Wi=14 and Re=0.01 was conducted in order to test the stability of the numerical scheme for large Weissenberg numbers. The results of the simulation are presented in Figure 9, which renders the magnitudes of the velocity field and extra stress tensor components. The streamlines that are computed using the LIC technique are overlayed onto the contour plots to validate the accurately converged solution of the velocity field and vortex shape. Afonso et al. [55], along with other similar studies such as Fernandes [11], Moreno et al. [54], Wittschieber et al. [56], report that high Weissenberg-number calculations can only be performed with the log-conformation technique, whereas standard stress formulation is systematically diverged. Since this study is based on the standard approach for the extra-stress tensor and has a goal to propose a Lagrangian reformulation for viscoelastic flows, it is not yet clear why the simulations of various point-cloud resolutions and high Wi numbers stay stable. What may be observed is that the Lagrangian CFL condition (22), which controls the adaptive time stepping, provides an adequate stability criterion that the PPE may yield the pressure field to keep simulations incompressible and stable.

The components of the extra stress tensor $\tau_{p}$ show that the viscoelastic stresses are extremely high in the horizontal direction, amplifying the narrow channel downstream. Around the sharp corner, all components of the tensor have a similar order of magnitude, while the gradients of the component values around the contraction are evident. This complex situation causes large viscoelastic accelerations in the fluid (through the term $\nabla \cdot \tau_{p}$) to flow around the corner. Consequently, the pressure gradient is extremely high at the corner to keep the flow incompressible. A slight upstream bending of streamlines may be seen at the corner lip, which is the result of these gradients. For smaller Weissenberg numbers, complete lip vortices are formed, as shown in Figure 7. We did not observe instabilities that were reported by some other numerical methods. Future work should also investigate the influence of shear bands on the vortices [58].

### 4.4. Die Swelling

**Figure 10:**
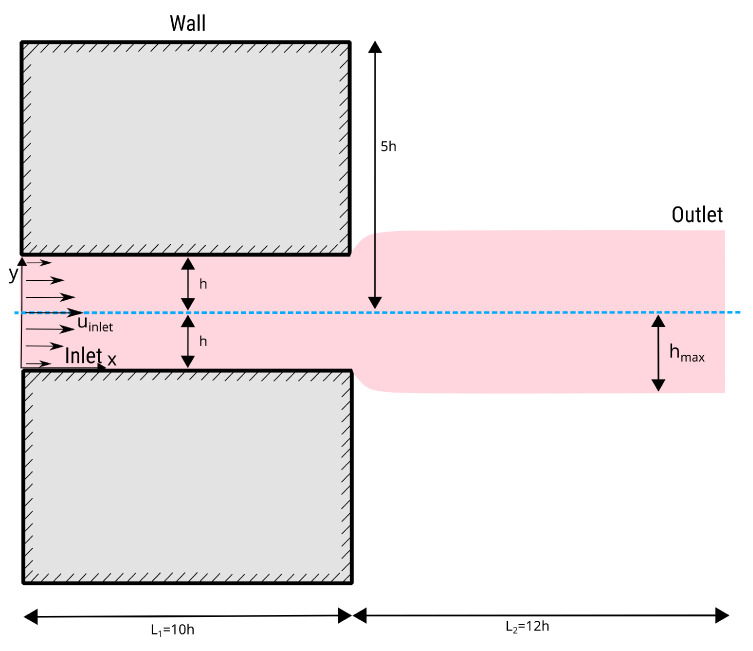
The computational domain for the die-swell experiment.

The phenomenon of the die swell, or the post-extrusion expansion of a polymer melt, has significance in the domain of polymer processing and serves as a critical benchmark for evaluating the proficiency of viscoelastic flow solvers. Due to its high sensitivity to constitutive modeling of viscoelastic properties, the die-swell problem is a good evaluator of solver accuracy. To represent the material’s response after exiting the die, the solver must incorporate the complexities of the stress–strain relationship, handling discontinuities in geometry and stress fields. The die-swell experiment is transient, but the swell ratio evolves until a steady state is reached [47,59]. Figure 10 depicts a schematic illustration of the numerical setup for the die-swell experiment in two dimensions. The whole domain is simulated considering a channel with a height of 2h and a length of 10h. The expansion zone is 12h long, with a total domain length of 22h. The dimensions of the swell domain are dimensioned in a way that boundary conditions for the inlet do not impact the flow near the channel exit.

**Figure 11:**
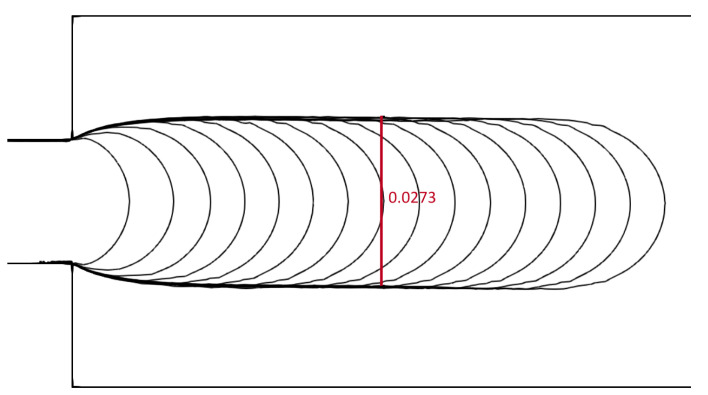
Snapshots of the free surface for the die-swell simulation, Wi=0.5 and Re=0.5. The evolution of the free surface (one contour per simulated second) is compared to the swell ratio in [47].

**Figure 12:**
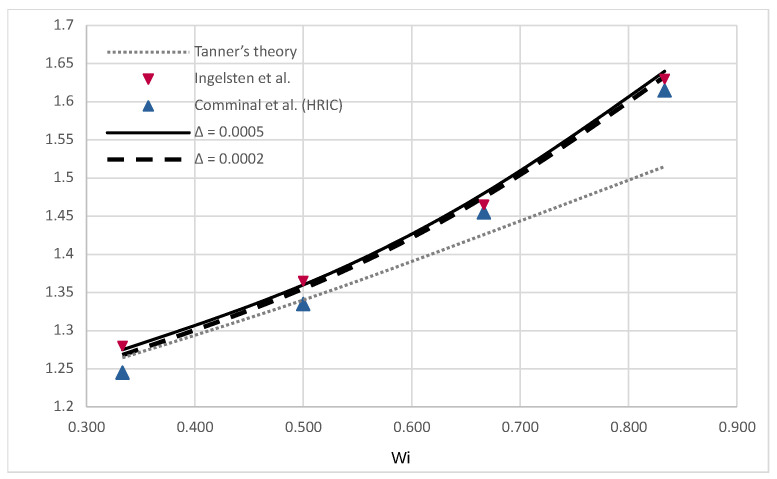
Comparison of the simulated evolution of the die swelling for various Weissenberg numbers between 0.333 and 0.833 [47,59].

**Figure 13:**
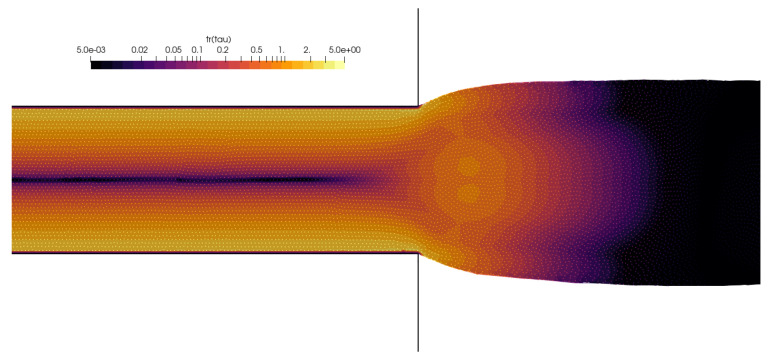
Contour plot of the instantaneous trace of the extra stress tensor for the die-swell simulation, Wi=0.5 and Re=0.5. Mesh-free points are also plotted to show the incompressible state of the fluid.

The main dimension was taken as h=0.01 m, and two initial point-spacing values were tested: Δ=0.0005 and Δ=0.0002. The evolution of the free surface, captured during the first 16 s of the simulation for Wi=0.5, is shown in Figure 11. The simulated swell corresponds to the numerically obtained results by Ingelsten et al. [47]. The simulations’ results are compared to Tanner’s theory and data from [59] and [47], and the comparison is presented in Figure 12. The swell ratio is calculated for the simulation results as $S_{r}=h_{max}/h$, i.e., the ratio of the swell width measured away from the channel and the channel width. To obtain a comprehensive understanding of the cases and numerical methods, please refer to the corresponding studies. The study by Comminal et al. [59] utilized an alternative definition of the Weissenberg number and swell ratio, which is acknowledged by Ingelsten et al. [47] and thus also included in Figure 12. Comminal et al. obtained disparate results when employing three subtly distinct numerical methods to simulate the identical flow. Nevertheless, the results of LDD simulations are in very good agreement with the referent data while slightly overpredicting compared to numerical methods for Wi>0.5. A contour plot of an instantaneous trace of the extra stress tensor is given in Figure 13, where it can be seen that the concentration of the extra stress is building up near the corners of the channel exit and in the center after the channel exit, while it diminishes after reaching a steady state.

## 5. Conclusions

This research addressed and resolved the current challenges of meshless Lagrangian methods for simulating viscoelastic materials, which opens up avenues for the optimization of industrial processes, the design of innovative materials, and the exploration of biological fluid dynamics.

Lagrangian differencing dynamics (LDD) is a Lagrangian method for incompressible flows, which has been thoroughly validated on complex Newtonian and non-Newtonian flows in complex domains. For this reason, it was taken as the baseline method to address the challenges of simulating viscoelastic materials in the Lagrangian context. A split-step scheme, or pressure Poisson reformulation of the Navier–Stokes equations, was introduced for incompressible viscoelastic flows in the Lagrangian context. The numerics based on LDD were introduced to solve the proposed scheme.

The main findings of this study highlight the LDD method’s effectiveness in accurately simulating viscoelastic flows, especially when combined with the Oldroyd-B model. The relaxation time and retardation time parameters in the Oldroyd-B model provide insights into the dynamic behavior of polymer solutions. The LDD method proves well-suited to simulating the behavior of viscoelastic materials, i.e., the meshless Lagrangian framework inherently captures large deformations and configurational memory effects that are hallmarks of viscoelasticity. The method’s applicability to real polymer fluid response has been demonstrated by successfully simulating benchmarks that are relevant to industrial polymer-processing scenarios. The lid-driven cavity test assessed LDD’s accuracy in capturing recirculation and shear-thinning in confined spaces. Droplet impact simulations evaluated its ability to handle complex fluid interfaces and behaviors during impact. The 4:1 sudden-contraction tests examined the performance in modeling flow separation, stress relaxation, and flow reformation in challenging geometries. The die-swell simulation tested LDD’s ability to replicate elongational viscosity and elastic effects during fluid extrusion. These benchmarks collectively confirm the new method’s effectiveness in predicting flow patterns and viscoelastic effects, aligning with experimental or theoretical expectations. Additionally, the method’s ability to handle complex geometries and free surfaces aligns well with the often intricate flow patterns observed in these materials.

The presented applications of the LDD method to viscoelastic materials pave the way for further exploration of complex flow phenomena in this material class. To extend the applicability of the presented findings, future work will explore alternative constitutive models beyond Oldroyd-B formulation, including investigations of the log-conformation approach to reach even higher Weissenberg numbers. Finally, while this study investigated derivative-based viscoelastic models in Lagrangian motion, future work will investigate integral formulations. Lagrangian mechanics tracks material points within a deforming body; therefore, it may benefit from the inherent memory effects captured by integral models.

## Data Availability

The original contributions presented in the study are included in the article, further inquiries can be directed to the corresponding author.

## Abbreviations

LDD	Lagrangian Differencing Dynamics
CFD	Computational Fluid Dynamics
ODE	Ordinary Differential Equation
PDE	Partial Differential Equation
HWNP	high Weissenberg Number Problem
SPH	Smoothed Particle Hydrodynamics
UCM	Upper-convected Maxwell
DNS	Direct Numerical Simulation
LST	Linear Stability Theory
NSE	Navier–Stokes equations
BDF2	Backward Differencing Formula of the Second Order
PSD	Principal Stress Difference
FIB	flow-induced Birefringence

## Appendix A. Divergence of the Diffusive Stress

The divergence of diffusion stress is scalar, presented in a fully expanded formulation:

$$\begin{array}{c} \nabla \cdot \left[\nabla \cdot \left(\mu \nabla \cdot u\right)\right]=\mu \frac{\partial }{\partial x}\left(\frac{\partial^{2}u}{\partial x^{2}}+\frac{\partial^{2}u}{\partial y^{2}}+\frac{\partial^{2}u}{\partial z^{2}}\right)+\\ \mu \frac{\partial }{\partial y}\left(\frac{\partial^{2}v}{\partial x^{2}}+\frac{\partial^{2}v}{\partial y^{2}}+\frac{\partial^{2}v}{\partial z^{2}}\right)+\\ \mu \frac{\partial }{\partial z}\left(\frac{\partial^{2}w}{\partial x^{2}}+\frac{\partial^{2}w}{\partial y^{2}}+\frac{\partial^{2}w}{\partial z^{2}}\right)+\\ \frac{\partial \mu }{\partial x}\left\{\left(\frac{\partial^{2}u}{\partial x^{2}}+\frac{\partial^{2}u}{\partial y^{2}}+\frac{\partial^{2}u}{\partial z^{2}}\right)+\frac{\partial^{2}u}{\partial x^{2}}+\frac{\partial^{2}v}{\partial y\partial x}+\frac{\partial^{2}w}{\partial z\partial x}\right\}+\\ \frac{\partial \mu }{\partial y}\left\{\left(\frac{\partial^{2}v}{\partial x^{2}}+\frac{\partial^{2}v}{\partial y^{2}}+\frac{\partial^{2}v}{\partial z^{2}}\right)+\frac{\partial^{2}u}{\partial y\partial x}+\frac{\partial^{2}v}{\partial y^{2}}+\frac{\partial^{2}w}{\partial z\partial y}\right\}+\\ \frac{\partial \mu }{\partial z}\left\{\left(\frac{\partial^{2}w}{\partial x^{2}}+\frac{\partial^{2}w}{\partial y^{2}}+\frac{\partial^{2}w}{\partial z^{2}}\right)+\frac{\partial^{2}u}{\partial z\partial x}+\frac{\partial^{2}v}{\partial z\partial y}+\frac{\partial^{2}w}{\partial z^{2}}\right\}+\\ \frac{\partial^{2}\mu }{\partial y\partial x}\left(\frac{\partial u}{\partial y}+\frac{\partial v}{\partial x}\right)+\frac{\partial^{2}\mu }{\partial z\partial x}\left(\frac{\partial u}{\partial z}+\frac{\partial w}{\partial x}\right)+\frac{\partial^{2}\mu }{\partial z\partial y}\left(\frac{\partial v}{\partial z}+\frac{\partial w}{\partial y}\right)+\frac{\partial^{2}\mu }{\partial x^{2}}\frac{\partial u}{\partial x}+\frac{\partial^{2}\mu }{\partial y^{2}}\frac{\partial v}{\partial y}+\frac{\partial^{2}\mu }{\partial z^{2}}\frac{\partial w}{\partial z}. \end{array}$$

The equation may be more cleanly written using vector and tensor notation:

(A1) $$\begin{array}{c} \nabla \cdot \left[\nabla \cdot \left(\mu \nabla \cdot u\right)\right]=\mu \;\nabla \cdot \left(\nabla^{2}u\right)+\nabla \mu \cdot \left[\nabla^{2}u+\nabla \cdot {\left(\nabla u\right)}^{T}\right]+{\langle H{\left(\mu \right)}^{T},\;\nabla u\rangle }_{F}, \end{array}$$

where the last term is known as the Frobenius inner product (or Hilbert–Schmidt inner product in more general contexts), defined as ${\langle A,\;B\rangle }_{F}=\mathrm{tr}\left\{A^{T}\;B\right\}$. It is defined as “the trace of the product of two tensors, where the first one is transposed”, or as “the sum of element-wise multiplication of two tensors”. $H\left(\mu \right)$ denotes the Hessian of μ.

To analyze the first term of Equation (A1), the curl–curl identity is defined as follows:

$$\nabla^{2}u=\nabla \left(\nabla \cdot u\right)-\nabla \times \left(\nabla \times u\right),$$

and since the curl operator always gives a solenoidal vector field, taking the divergence of the above equation leads to the equivalence:

$$\nabla \cdot \left(\nabla^{2}u\right)=\nabla^{2}\left(\nabla \cdot u\right).$$

The approximation of Equation (A1) is summarized as follows:

(A2) $$\nabla \cdot \left[\nabla \cdot \left(\mu \nabla \cdot u\right)\right]=2\mu \;\nabla^{2}\left(\nabla \cdot u\right)+2\nabla \mu \cdot \nabla^{2}u+2\nabla^{2}\mu \;\left(\nabla \cdot u\right),$$

where the first term is dominant to be included as the source term for the pressure, to suppress compressibility in highly viscous fluid due to diffusive spreading of the velocity divergence.

## Appendix B. Divergence of the Divergence of the Extra Stress

An arbitrary symmetric second-rank tensor in three dimensions is defined as a 3 × 3 matrix:

$$T=\left[\begin{array}{ccc} T_{11} & T_{12} & T_{13}\\ T_{12} & T_{21} & T_{23}\\ T_{13} & T_{23} & T_{33} \end{array}\right].$$

In the context of viscoelastic fluid flow, all elements of the matrix are dependent on position in space and time, $T_{ij}=f\left(x,\;t\right)$. The “divergence of the tensor divergence”, $\nabla \cdot \left(\nabla \cdot T\right)$, is scalar:

(A3) $$\begin{array}{cc} \nabla \cdot \left(\nabla \cdot T\right) & =\frac{\partial^{2}T_{11}}{\partial x^{2}}+\frac{\partial^{2}T_{22}}{\partial y^{2}}+\frac{\partial^{2}T_{33}}{\partial z^{2}}+2\frac{\partial^{2}T_{12}}{\partial x\partial y}+2\frac{\partial^{2}T_{13}}{\partial x\partial z}+2\frac{\partial^{2}T_{23}}{\partial y\partial z}. \end{array}$$

Alternatively, the above expression (A3) may be written using the tensor operation:

(A4) $$\nabla \cdot \left(\nabla \cdot T\right)={\langle H,\;T\rangle }_{F},$$

where the operation on the right-hand side of Equation (A4) is known as the Frobenius inner product, ${\langle A,\;B\rangle }_{F}=\mathrm{tr}\left\{A^{⊤}\;B\right\}$. It is formally defined as “the trace of the product of two tensors, where the first one is transposed”, whereas for understanding of the above, it is more convenient to define it as “the sum of element-wise multiplication of two tensors”. H is the Hessian operator, $H=\partial^{2}/\left(\partial x_{i}\partial x_{j}\right)$.

Since the above equation is not easy to interpret numerically, a simplification in numerical modeling is needed. The Hessian of a scalar is a symmetric tensor due to the symmetry of second derivatives, and it is also defined as the “transpose of the Jacobian matrix of the gradient of some function”, $H\left(f\right)=J{\left(\nabla f\right)}^{⊤}$. Furthermore, if one takes that the tensor T is based on a vector gradient, T=∇v, where $v={\left[u\;v\;w\right]}^{⊤}$, then the tensor is defined as follows:

$$T=\left[\begin{array}{ccc} \frac{\partial u}{\partial x} & \frac{\partial u}{\partial y} & \frac{\partial u}{\partial z}\\ \frac{\partial v}{\partial x} & \frac{\partial v}{\partial y} & \frac{\partial v}{\partial z}\\ \frac{\partial w}{\partial x} & \frac{\partial w}{\partial y} & \frac{\partial w}{\partial z} \end{array}\right].$$

It follows that the divergence of the tensor divergence is as follows:

(A5) $$\begin{array}{cc} \nabla \cdot \left(\nabla \cdot T\right) & =\left(\frac{\partial^{3}u}{\partial x^{3}}+\frac{\partial^{3}u}{\partial y^{2}\partial x}+\frac{\partial^{3}u}{\partial z^{2}\partial x}\right)+\\ \; & +\left(\frac{\partial^{3}v}{\partial y^{3}}+\frac{\partial^{3}v}{\partial y\partial x^{2}}+\frac{\partial^{3}v}{\partial z^{2}\partial y}\right)+\\ \; & +\left(\frac{\partial^{3}w}{\partial z^{3}}+\frac{\partial^{3}w}{\partial z\partial x^{2}}+\frac{\partial^{3}w}{\partial z\partial y^{2}}\right)\\ \; & =\nabla^{2}\left(\frac{\partial u}{\partial x}+\frac{\partial v}{\partial y}+\frac{\partial w}{\partial z}\right)\\ \; & =\nabla^{2}\left(\nabla \cdot v\right)\\ \; & =\nabla^{2}\left[\mathrm{tr}\left(T\right)\right]. \end{array}$$

In other words, if the tensor arises as the gradient of some vector field, div–div operation on the tensor boils down to the Laplacian of its trace. It is straightforward to show that the expression also holds for $T={\left(\nabla v\right)}^{T}$ and for tensors that are arising from symmetrized vector gradients, e.g., T=12∇v+∇vT. In the context of the extra stress tensor, its trace $\mathrm{tr}\left(\tau_{p}\right)$, represents the isotropic part of the stress that contributes to volumetric changes in the material. It is a measure of the normal stresses that implies a tendency for volumetric expansion or contraction under the stress conditions. In summary, the pressure equation is based on the Laplacian of the pressure; therefore, in a numerical context, it makes sense to use Equation (A5) as the source term for the pressure due to the elasticity in the fluid.
